# Longitudinal anellome dynamics in the upper respiratory tract of children with acute respiratory tract infections

**DOI:** 10.1093/ve/vead045

**Published:** 2023-07-13

**Authors:** Le Cao, Yingying Ma, Zhenzhou Wan, Bing Li, Weimin Tian, Chiyu Zhang, Yanpeng Li

**Affiliations:** Shanghai Public Health Clinical Center, Fudan University, 2901 Caolang Road, Jinshan District, Shanghai 201508, China; Shanghai Public Health Clinical Center, Fudan University, 2901 Caolang Road, Jinshan District, Shanghai 201508, China; Medical Laboratory of Taizhou Fourth People’s Hospital, 99 North Gulou Road, Taizhou 225300, China; Shanghai Public Health Clinical Center, Fudan University, 2901 Caolang Road, Jinshan District, Shanghai 201508, China; Shanghai Public Health Clinical Center, Fudan University, 2901 Caolang Road, Jinshan District, Shanghai 201508, China; Shanghai Public Health Clinical Center, Fudan University, 2901 Caolang Road, Jinshan District, Shanghai 201508, China; Shanghai Public Health Clinical Center, Fudan University, 2901 Caolang Road, Jinshan District, Shanghai 201508, China

**Keywords:** anellovirus, ARTI, children, metagenomics, evolution

## Abstract

Anelloviruses (AVs) are ubiquitous in humans and are the most abundant components of the commensal virome. Previous studies on the diversity, transmission, and persistence of AVs mainly focused on the blood or transplanted tissues from adults; however, the profile of the anellome in the respiratory tract in children are barely known. We investigated the anellome profile and their dynamics in the upper respiratory tract from a cohort of children with acute respiratory tract infections (ARTIs). Different to that in adult, *betatorquevirus* is the most abundant genus, followed by *alphatorquevirus*. We found that the relative abundance of *betatorquevirus* was higher in earlier time points, and in contrast, the abundance of *alphatorquevirus* was higher in later time points; these results might suggest that *betatorquevirus* decreased with age and *alphatorquevirus* increased with age in childhood. No difference regarding the diversity and abundance of anellome was found between single and multiple ARTIs, consistent with the idea that AV is not associated with certain disease. Most AVs are transient, and a small proportion (8 per cent) of them were found to be possibly persistent, with persistence time ranging from 1 month to as long as 56 months. Furthermore, the individual respiratory anellome appeared to be unique and dynamic, and the replacement of existing AVs with new ones are common over different time points. These findings demonstrate that *betatorquevirus* may be the early colonizer in children, and the individual respiratory anellome is unique, which are featured by both chronic infections and AV community replacement.

## Introduction

Anelloviruses (AVs) are one of the most abundant eukaryotic virus components of the human virome, which have a circular, single-stranded DNA genome ranging from 2.0 to 3.9 kb in length (Spandole et al. 2015; Kaczorowska and van der Hoek 2020). Even though human AV was first identified in 1997 in a hepatitis patient (Nishizawa et al. 1997), no clear association of this virus with any diseases was found during the subsequent studies (Webb, Rakibuzzaman, and Ramamoorthy 2020; Taylor et al. 2022). AVs are ubiquitous among humans, and the positivity rates of AVs could approach 90 per cent in certain population (Spandole et al. 2015), and AVs could be detected in different organs, tissues, and biological samples, including blood, feces, genital tract, lymph nodes, liver, lung, bone marrow, saliva, urine, respiratory swabs, skin, etc. (Kumata et al. 2020). In addition, AVs are also highly divergent, with limited genetic identities being shared between different species, and the presence of AVs is featured by the co-infection with multiple genotypes (Ball et al. 1999; Khudyakov et al. 2000; Niel, Saback, and Lampe 2000; Arze et al. 2021).

Even though there is a lack of AV pathogenicity, the infection of AVs is believed to be controlled by the host immune systems, and its presence is considered as a reflection of the host immune status (Touinssi et al. 2001; Hino and Miyata 2007; Davidson and Shulman 2008; Focosi et al. 2016; Rezahosseini et al. 2019; Redondo et al. 2022). For example, compared to healthy people, higher abundance of AVs was found in conditions such as organ transplantation (van Rijn Al et al. 2023; Zeng et al. 2023), HIV-1 and/or hepatitis C virus (HCV) infection (Liu et al. 2021; Li et al. 2022), cancer (Zhong et al. 2006; Li et al. 2023), as well as inflammation (Kaelin et al. 2022). Previous studies have indicated that AVs could be infected during early childhood (Bagaglio et al. 2002; Liang et al. 2020; Väisänen et al. 2022), and persistent or chronic infection by the same variants may occur throughout life (Kaczorowska et al. 2022b). Interestingly, Kaczorowska et al. investigated the prevalence of anellome in the early stage of life and reported that *betatorquevirus* and *gammatorquevirus* were frequently detected in early childhood (younger than 12 months), while the prevalence of *alphatorquevirus* increased in children older than 12 months (Kaczorowska et al. 2022a). In addition, it is also hypothesized that the composition of the anellome in the population is continuously changing probably due to the clearance and/or evolution of existing strains and the reinfection of new strains (Maggi et al. 2001; Kaczorowska and van der Hoek 2020).

Acute respiratory tract infections (ARTIs) are one of the most common cause of disease in children, which account for about 4 million annual deaths worldwide (Tregoning and Schwarze 2010). Even though AVs are not the cause of ARTIs, they are often detected from the patients’ respiratory samples. Airway is believed to be a critical transmission route of AVs (Maggi et al. 2003; Kaczorowska and van der Hoek 2020), and AVs might be associated with some respiratory disorders in children (Pifferi et al. 2006; Freer et al. 2018; Dodi et al. 2021; Bal et al. 2022). However, most studies regarding the transmission, evolution, and persistence of AVs mainly focused on the blood, which has the highest viral load and diversity of AVs. However, little is known about the anellome profile in children’s respiratory tract, and several major questions remain concerning how the respiratory anellome persists, whether specific AVs are shared and transmitted through airway and are associated with respiratory disease. In this study, we analyzed the respiratory anellome in children with ARTIs and investigated the stability and dynamics of AV infections over time.

## Materials and methods

### Study subjects

To explore the respiratory anellome and its dynamics in children with ARTIs, we selected the cohort from our previous study for further analysis (Li et al. 2019). During the previous study, we screened 4,407 children diagnosed as having ARTI from 2009 to 2015 in Shanghai, China. Children were identified as Single-ARTI group (only one ARTI episode was captured during the study window) and Multiple-ARTI group who experienced two or more episodes of ARTIs (herein defined as multiple ARTIs). In total, this cohort contained forty-eight children with single ARTI episode (single ARTI) and sixty-one children with multiple ARTI episodes (multiple ARTIs). Among the sixty-one children with multiple ARTIs, twenty-nine children had two ARTI episodes, twenty-three had three episodes, nine had four episodes. Viral metagenomics (enrichment of encapsidated DNA and RNA viruses) was performed on the nasopharyngeal swabs collected from each child. The median age of Single ARTI group was 40.8 (4.8–63.6) months, median age of the first, second, third, and fourth episodes from Multiple ARTI group were 45.6 (range, 9.6–114), 52 (12–122), 72 (28–142), and 60.5 (41–97) months, respectively. The detailed definition and selection criteria of single and multiple ARTIs are described in our previous study. Common viruses previously detected from these respiratory samples included human rhinovirus (24.1 per cent), influenza virus (19.8 per cent), enterovirus (15.6 per cent), herpesvirus (15.1 per cent), coronavirus OC43/NL63/229E (10.8 per cent), parainfluenzavirus (9.4 per cent), respiratory syncytial virus (7.1 per cent), adenovirus (7.1 per cent), papillomavirus (3.8 per cent), and metapneumovirus (2.8 per cent). In order to further focus on the anellome from the respiratory tract, all the metagenomic sequencing data of these children were downloaded from China National GeneBank Sequence Archive (https://db.cngb.org/cnsa/) of China National GeneBank DataBase (project number CNP0000429) and were subject to anellome analyses.

### Nucleic acid extraction, amplification, and sequencing

The detailed protocol for the metagenomic sequencing was described in detail in our previous study (Li et al. 2019). In brief, encapsidated viruses were enriched through sequential steps including centrifugation, filtration, and a cocktail of nucleases treatment. Both DNA and RNA from the viruses were extracted using QIAamp MinElute Virus Kit (Qiagen, Germany), and the total nucleic acids were amplified using a random-amplification approach (REPLI-g Single Cell WTA Kit, Qiagen, Germany). The libraries were sequenced on BGI-Seq500 platform.

### Anellome analyses

Metagenomics data were analyzed as previously described (Li et al. 2023, 2022). Briefly, raw data were first filtered by Trimmomatic v.0.38 (Bolger, Lohse, and Usadel 2014) by removing adaptors and low-quality sequences. Reads of human origin were subtracted from the data using Bowtie2 v.2.3.4.3 (using hg38 database) (Langmead and Salzberg 2012). Then, the remaining high-quality reads were de novo assembled using Megahit v.1.1.3 (Li et al. 2015). Assembled contigs were then mapped against the viral nucleic acid and protein database using BLAST 2.11.0+ (*E* < 10^–10^) and BLASTx (*E* < 10^–5^) (DIAMOND v.0.9.24) (Buchfink, Xie, and Huson 2015). All the viral hit candidates were searched against the National Center for Biotechnology Information (NCBI) nucleotide (NT) and nonredundant protein database to further identify and remove sequences that have higher similarity to non-viral sequences.

Only the contigs assigned to AV were selected for further subsequent analysis. All the AV contigs based on NCBI Taxonomy annotation were further curated using International Committee on Taxonomy of Viruses (ICTV) AV references (https://ictv.global/taxonomy) (Varsani et al. 2021). AV contigs were classified as different viral lineages (AV taxonomic unit, ATU) using an in-house pipeline. Briefly, referring to the previous study (Arze et al. 2021), we set the 2.5 per cent NT sequence identity and 70 per cent coverage for the shorter sequence as the cutoff value for lineages using CD-hit v4.8.1 (Fu et al. 2012; Arze et al. 2021). Using this standard, all AV contigs were assigned into 1,352 clusters. According to the CD-hit algorithm, the longest contig is the representative sequence in each cluster. Next, we retained those clusters with the representative contig lengths greater than 500 bp. This resulted in a total number of 487 clusters for subsequent analysis. All the AV reads/contigs were available upon kind request.

### Phylogenetic analysis of ORF1 region of anellovirus

The ORF1 region of AV was extracted using NCBI’s ORF Finder tool (https://www.ncbi.nlm.nih.gov/orffinder/) using the ‘any sense codon’ option, which were then curated by aligning against an in-house AV reference sequence database. Viral nucleic acid sequences were first translated into amino acids and aligned using MAFFT (E-INS algorithm) (Katoh, Rozewicki, and Yamada 2019). Only sequences longer than 1,000 bp were used for phylogenetic analysis. Model test program was used to determine the best substitution model. Phylogenetic trees were inferenced using the maximum likelihood method with IQ-Tree (Minh et al. 2020). Phylogenetic trees based on NT sequences were generated using the bootstrap method (1,000 times) under a GTR + F + R6 model. All ATU sequences used in the phylogenetic tree analysis were deposited in the GenBank under accession number OQ971812-OQ971862.

### Statistical analysis

The alpha diversity was calculated by the Shannon diversity index, which measures species diversity and relative abundance, and richness score, which computes the number of species in a community. Viral abundance was shown as reads per million (RPM). Continuous variables between groups were compared by the nonparametric Mann-Whitney U test or Kruskal-Wallis test with Dunn’s correction. A difference with *P* < 0.05 was considered as statistically significant.

## Results

All the metagenomic sequencing data were subjected to virus annotation as described in Materials and methods, and twenty-five children with single ARTI and forty-four children with multiple ARTIs (including nineteen children who had two ARTI episodes, sixteen who had three, and nine who had four episodes) were positive for AVs. The percentage of AV-positive children was 63.3 per cent (69/109). The median of age of AV-positive children was not different from the AV-negative ones (49.2 vs. 50.4 months). Using previously described respiratory virus profiles in these samples (see Materials and methods), we further compared the distribution of these respiratory viruses between AV-positive and -negative children. AV-positive children had higher positive rates for nine of ten common respiratory viruses (Figure S1A). However, we did not find specific association (co-occurrence rate) between AVs and specific respiratory viruses (Figure S1B).

**Figure 1.**
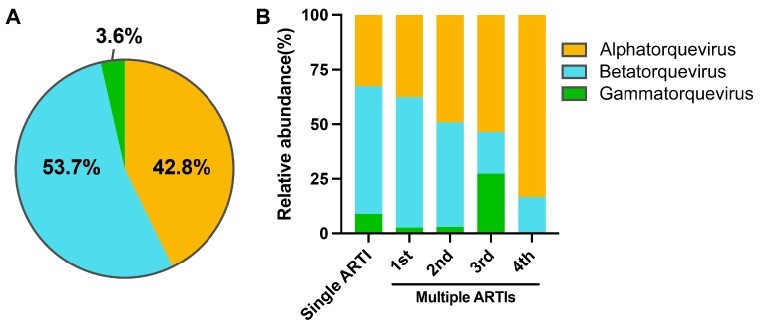
The profile of *Anelloviridae* in upper respiratory tract of children with ARTIs. (A) The pie chart shows the overall proportion of *alphatorquevirus, betatorquevirus*, and *gammatorquevirus* in this cohort. (B) The relative abundance of *alphatorquevirus, betatorquevirus*, and *gammatorquevirus* among single and multiple ARTI groups.

Among the respiratory anellome in children, *betatorquevirus* (53.7 per cent) was the most dominant genus, followed by *alphatorquevirus* (42.8 per cent), and *gammatorquevirus* only accounted for 3.6 per cent of the total anellome (Fig. 1A). Next, we compared the relative abundance of each genus of AVs in single and multiple ARTIs, and no significant difference was found between the two groups. However, for the different episodes of multiple ARTIs, there was an increasing trend of the relative abundance of *alphatorquevirus* and a decrease of *betatorquevirus* at later ARTI episodes as compared to the earlier ones (Fig. 1B), but no significance was detected. Next, to minimize the age variations between individuals, we selected those children who had two or more sampling points positive for anelloviridae and analyzed the changes of the AV genus with age within each child (Figure S2). We found that in most of the children, the relative abundance of *betatorquevirus* decreases (*P* = 0.03), while that of *alphatorquevirus* increases with age (*P* = 0.04), even though a couple of data points showed an opposite trend.

**Figure 2.**
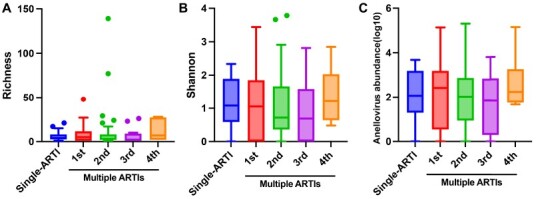
The anellovirus ATU diversity among different groups. Comparison of the richness (A), Shannon (B), and abundance (C) of anellovirus between single and multiple ARTI groups. Alpha diversity and abundance was calculated at the anellovirus lineages (ATUs) level. The comparisons were performed using the Kruskal-Wallis test with Dunn’s corrections.

To investigate the burden and complexity of the respiratory AVs in single and multiple ARTIs, we compared the viral abundance (RPM) and viral diversity (Richness and Shannon indexes) from different groups (Figure S3). There was no significant difference regarding the abundance and diversity of AVs between different groups. The genomic sequences of AVs are highly divergent, and it is difficult to accurately estimate their community diversity only based on the annotations resulting from the blast alignment. Thus, each AV contig (over 500 bp) was further determined as different AV lineages (ATU) as described in Materials and methods. In total, 487 ATUs were defined from all the children. Similar to the above result, no significant difference of the ATU diversity was found between different groups (Fig. 2).

**Figure 3.**
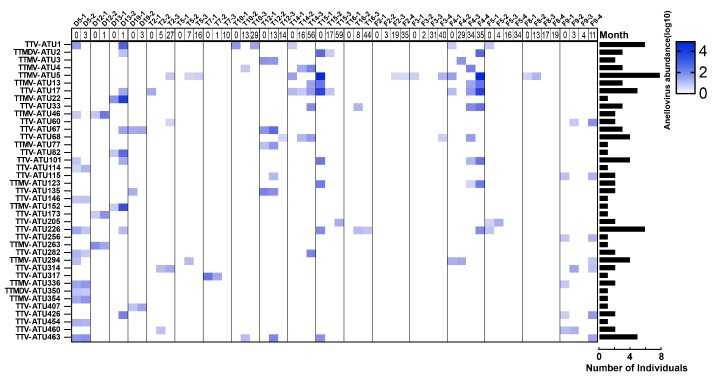
Persistent infection of various anellovirus lineages (ATUs) over different episodes of multiple ARTIs. Heatmap shows the presence and relative abundance of each ATU. The histogram on the right shows the prevalence of each ATU in all the samples. The episode names and their sampling time intervals are shown at the top of the heatmap. Persistent ATUs are detected in eighteen children, and all the ATUs from these eighteen children are shown in Table S1.

Persistent infections of AVs in the blood were reported (Arze et al. 2021; Kaczorowska et al. 2022b). However, how AVs persist in the upper respiratory tract is unknown. Using the longitudinal samples from the children with multiple ARTIs, we investigated the persistent infections of AVs in children’s upper respiratory tract using the ATU classification. In total, 39 ATUs (8 per cent of the total ATUs) were determined as persistent AVs (determined by their detection at different time points) in eighteen children (Figure S4A). The profiles of the AV persistence in the upper respiratory tract were highly individual-specific; for example, persistent infection was detected in 40.9 per cent (18/44) of the children with multiple ARTIs, and the number of persistent ATUs ranged from one (nine children) or two (two children, #D19 and #T19) to seven (one child, #F4) or nine (one child, #D5) in different individuals (Fig. 3, Table S1). Besides, the persistence time of different AVs varied greatly between different individuals; some ATUs persisted for only 1 month, while other ATUs could persist up to 56 months (#T14).

**Figure 4.**
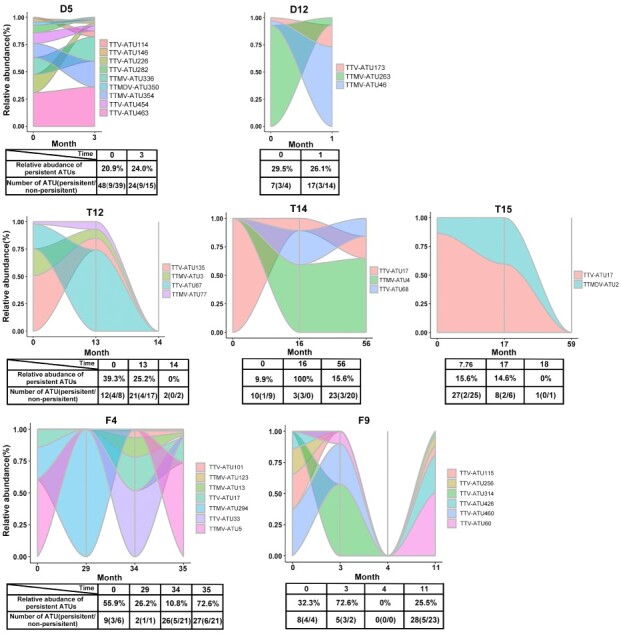
Dynamic changes of persistent ATUs over longitudinal time points. Each tide graph indicates the changes of the relative abundance of persistent ATUs. The number of persistent (ATUs that were present in more than two sampling time points)/nonpersistent ATUs, as well as the relative abundance of persistent ATUs to all the ATUs at each time point is shown in the table at the bottom of each graph. Due to the space limit, only two individuals (D5 and D12) with two ARTI episodes, three individuals (T12, T14, and T15) with three episodes, and two individuals (F4 and F9) with four episodes from multiple ARTI are shown, and the results of all the other children are displayed in Table S1. The first line shows the results for children with two episodes, second line for children with three episodes, and third line for children with four episodes from multiple ARTI group.

Next, we further analyzed the constitutions and dynamics of the respiratory anellome over different time points. The ATU profiles were highly variable at different sampling time points as well as between different individuals regarding the ATU types, the number of ATUs, and persistent ATUs (Fig. 4, Table S1). An average of 10.6 ATUs (range, 0–139) were detected in all the episodes of multiple ARTIs, and persistent ATUs accounted for an average relative abundance of 26.4 per cent (range, 0–100 per cent) (Table S1). For example, in children with two ARTI episodes, the types of persistent ATUs and their relative abundance in D5 was relatively stable between the two episodes over 3 months, and the dominant ATUs stayed the same. However, in D12, three persistent ATUs were detected, and the dominant ATU was replaced by the other two ATUs after 1 month. In children with three ARTI episodes, four (T12) and two (T15) ATUs persisted for more than 1 year, and no persistent ATUs could be detected at the last episode. In T14, the first episode was dominated by one ATU (ATU17), and two more ATUs emerged after 16 months. These three ATUs persisted up to 56 months. Some dominant ATUs could be the same in different individuals (such as ATU 17 in both T14 and T15), and certain dominant ATU (ATU4 in T14) could persist for up to 40 months. In children with four ARTI episodes (F4 and F9), the anellome profiles and the dominant ATUs were ever changing in each episode. In F4, several persistent ATUs were detected at the first episode, then they were absent in the second episode (month 29) and re-emerged thereafter (month 34 and 35). In F9, similar phenomenon was observed that all the ATUs from the second episode were absent in the third episode (month 4) and re-emerged at the fourth episode (month 11).

In addition to the persistent ATUs, ATUs that were detected only once (nonpersistent) were also highly dynamic over different ARTI episodes and among different individuals (Fig. 4, Table S1). We observed that the nonpersistent AV community was continuously changing, which may be due to the clearance and/or evolution of existing ATUs and the reinfections of new ones. In total, an average of 8.8 ATUs (range, 0–136) were detected only once (disappeared or emerged) at each episode. To give an example, after 3 months, as much as thirty-nine ATUs disappeared at the second ARTI episode in D5, which was accompanied by the emergence of fifteen different ATUs. Seven ATUs from the first episode of F9 were replaced by four new ATUs at the second episode, then all ATUs disappeared at the third episode and no ATUs emerged at this time point; 7 months later, twenty-three new ATUs emerged at the fourth episode (Fig. 4 and Table S1).

**Figure 5.**
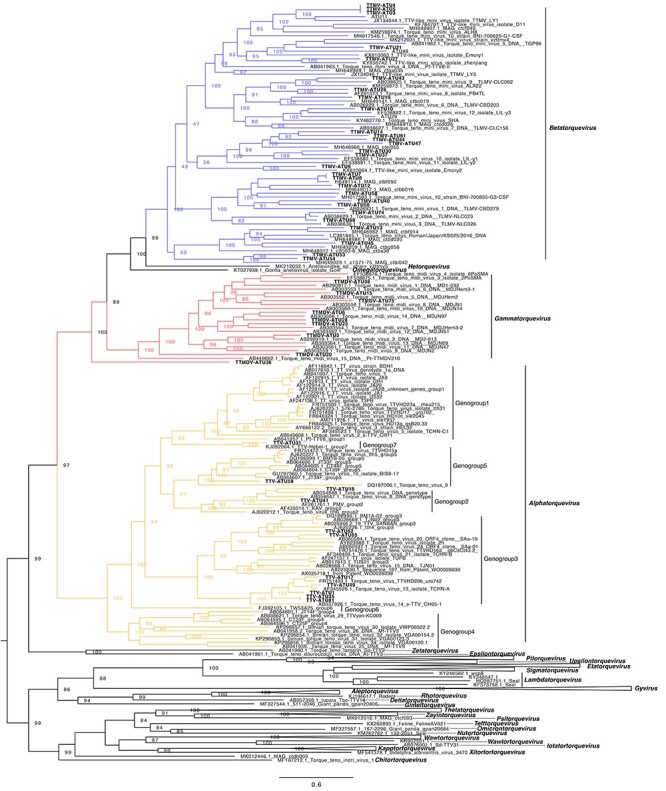
Maximum likelihood phylogenetic tree of the anellovirus lineages (ATUs). The red asterisks indicate anellovirus sequences identified in this study. The different colors represent for the three genera of anellovirus. All the anellovirus references from ICTV were included for this analysis, and the main genogroups of *alphatorquevirus* were labeled.

In order to determine the evolutionary relationship of these AVs, phylogenetic analysis was performed using the ATUs longer than 1000 bp and all the reference AV sequences from ICTV (Fig. 5). All the ATUs clustered into the three main AV genera (*alpha-, beta*-, and *gammatorquevirus*). Most of the ATUs (60.8 per cent) clustered with AV sequences from *betatorquevirus*. Further analysis showed an average genetic identity of 93.5 per cent (range, 79.5–100 per cent) of these ATUs to existing AVs in the current database, which means that there were no divergent or novel AV species circulating in the respiratory tract of these children.

## Discussion

In this study, we analyzed the upper respiratory anellome from a cohort of children with single or multiple ARTIs. In our previous study, no difference in the positive rate of AVs was found between single and multiple ARTIs (Li et al. 2019). Here we found no difference regarding the abundance and diversity of AVs between different groups, which suggests that AVs are not associated with multiple ARTIs. About 63 per cent of the children in this cohort were positive for AVs, similar to the AV prevalence in upper respiratory samples of pediatric patients with fever or ARTI (McElvania TeKippe et al. 2012; Bal et al. 2022). Higher rate of other common respiratory virus infections was found in AV-positive individuals, while no age differences were found between AV-positive and -negative individuals, indicating that the presence of AV in children’s respiratory tract could be more related to their health conditions and immune states.

AVs could be found in multiple sample types, and most studies have shown that *alphatorquevirus* is the dominant AV genus of the blood anellome in adults (Spandole-Dinu et al. 2018; Thijssen et al. 2020; Cebriá-Mendoza et al. 2021; Liu et al. 2021; Kaczorowska et al. 2022b; Li et al. 2022). However, based on the results from a few studies focusing on children (McElvania TeKippe et al. 2012; Cordey et al. 2021; Kaczorowska et al. 2022a; Rivera-Gutiérrez et al. 2023), *betatorquevirus* was the most frequent genus in the blood and nasopharyngeal samples, and *betatorquevirus* was proposed to be the pioneer AV population during the early childhood. Even though our data indicated a similar phenomenon that *betatorquevirus* rather than *alphatorquevirus* is the most abundant genus in children, and their relative abundance may be reversed with age, however, only a weak trend could be observed in this cohort. This may be due to a relative older age of our cohort than previous studies, and the dominance of *betatorquevirus* was not significant. Still, these data further indicated that *betatorquevirus* may be the main AVs that early colonize in children, and *alphatorquevirus* gradually steps in and becomes the dominant genus as observed in adults (Kaczorowska et al. 2022a). AVs were shown to be monitored and controlled by our immune system (Touinssi et al. 2001; Hino and Miyata 2007), and the replacement of *betatorquevirus* by *alphatorquevirus* may be due to its higher fitness to our immune system. However, this hypothesis needs evidence from future immunological and virological experiments.

Chronic infections of AVs were reported mainly in the human blood (Jazaeri Farsani et al. 2013; Bédarida et al. 2017; Arze et al. 2021; Kaczorowska et al. 2022b). In this study, we demonstrated that AVs could also persist in children’s respiratory tract. Even though we observed that specific ATUs could persist as long as 4–5 years in some individuals, individual anellome profile appeared to be highly unique, and there was no core anellome that were shared among different children. It was previously estimated that over 10^10^ AV virions are generated per day, and most of the AVs in plasma could be cleared and replenished every day (Maggi and Bendinelli 2010), suggesting the clearance/reinfection of AVs. In line with this hypothesis, we also observed that only a small proportion of the ATUs (8 per cent) showed persistence, and the vast majority only existed once across the longitudinal time points. However, due to their high mutation rates and extensive recombination events (Spandole et al. 2015; Arze et al. 2021), the genomic sequences of existing AVs could change substantially, and the detection of new AVs at later time points might be the result of the accumulated mutations of previous AVs rather than the clearance and reinfection of new ones. Furthermore, the negative result of specific ATUs may reflect the absence of them or it may also represent a window when they are below the detection limit of the detection method. Thus, it is difficult to estimate the actual duration time, AV types, as well as to what extent they could persist and/or reinfect. It was shown that AVs could transmit via the respiratory airway (Spandole et al. 2015; Kaczorowska and van der Hoek 2020). We found that the same ATUs could be detected in different children, which indicated possible transmission of AVs through the airway among this cohort. Even though there were no direct evidence that children in this cohort had reported transmission events between each other, most of them lived in the same or nearby communities and visited the same hospital for ARTI events. Thus, it is highly possible that the shared AVs were the result of airway transmissions.

AVs can be detected from different tissues and body compartments. Previous studies from lung-transplanted patients have shown that certain AVs were transmitted and shared by bronchoalveolar lavage fluid and plasma (Segura-Wang et al. 2018; Abbas et al. 2019), while other study suggests a compartment-specific AV dynamics (Widder et al. 2022). Thus, it would be interesting to analyze in future how the AVs are shared and communicated in different body compartments, such as the respiratory tract, blood, feces, and tissues, and whether some AV species or ATUs are tissue-specific. Answering these questions not only helps to understand the basic biological characteristics of AVs, it may also extend our knowledge of the potential translational use of AV. For example, in addition to its potential use as a biomarker for the host immune state, recent studies have suggested that AV could be manipulated as a gene therapy vector considering its symbiotic relationship with human, low immunogenicity, and reinfection capacity (Arze et al. 2021; Nawandar et al. 2022; Venkataraman et al. 2022). Thus, the discovery of tissue-specific AVs in respiratory tract or other body compartments with long persistence may be considered as potential candidates in future vector design.

This study has several limitations. First, only ARTI children and a few time points were available and investigated, and the detection of the same ATUs over 2–3 time points may also be the result of reinfection of the same ATU rather than persistence. Thus, more sampling points including the control samples from healthy conditions are needed to accurately reflect the dynamics of anellome. The healthy children is an important group to be included in future works as it could help to clarify the impact of AV on ARTI and/or the impact of ARTI on AV. Second, most of the children in this study were over 3 years old, and their anellome in the early life was not clear. A follow-up study with longitudinal samples from newborns to childhood could further reveal how does the anellome colonize and evolve thereafter, whether *betatorquevirus* is the very first virus after delivery, and how does the *alphatorquevirus* become the dominant genus.

In conclusion, we characterized the anellome of the upper respiratory tract from a cohort of ARTI children. The data demonstrate that the individual respiratory anellome is unique, with *betatorquevirus* being the possible early colonizer in children. Most AVs are transient, and the replacement of existing AVs with new ones are common. Some AVs may persist up to 5 years in the respiratory tract.

## Supplementary Material

vead045_Supp
