# Supplementary material for: Longitudinal anellome dynamics in the upper respiratory tract of children with acute respiratory tract infections
Source: Virus Evol. 2023 Jul 13;9(2):vead045. doi: 10.1093/ve/vead045 (PMC10478798; doi:10.1093/ve/vead045)
Supplement: vead045_Supp [file vead045_supp.zip › suppl_data/Supplementary figures.docx]

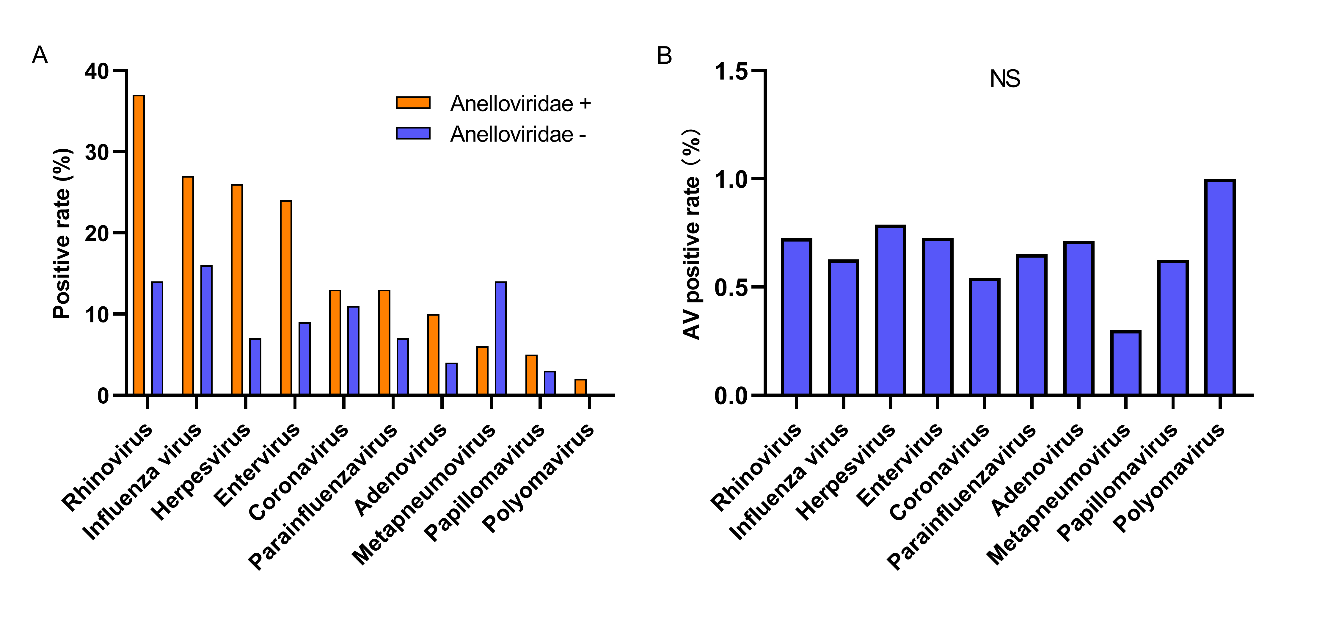


**Figure S1. The prevalence of common respiratory viruses in anellovirus positive or negative samples.** Figure S1A. All the samples were divided into anellovirus positive or negative groups, and the positive rates of rhinovirus, influenza virus, herpesvirus, enterovirus, coronavirus, parainfluenzavirus, adenovirus, metapneumovirus, papillomavirus and polyomavirus were shown for each group. Figure S1B. Possible association (co-occurrence rate) of anellovirus with each different common respiratory virus was shown. The co-occurrence rate was comparable among different viruses. ns, not significant (Fisher’s exact test).


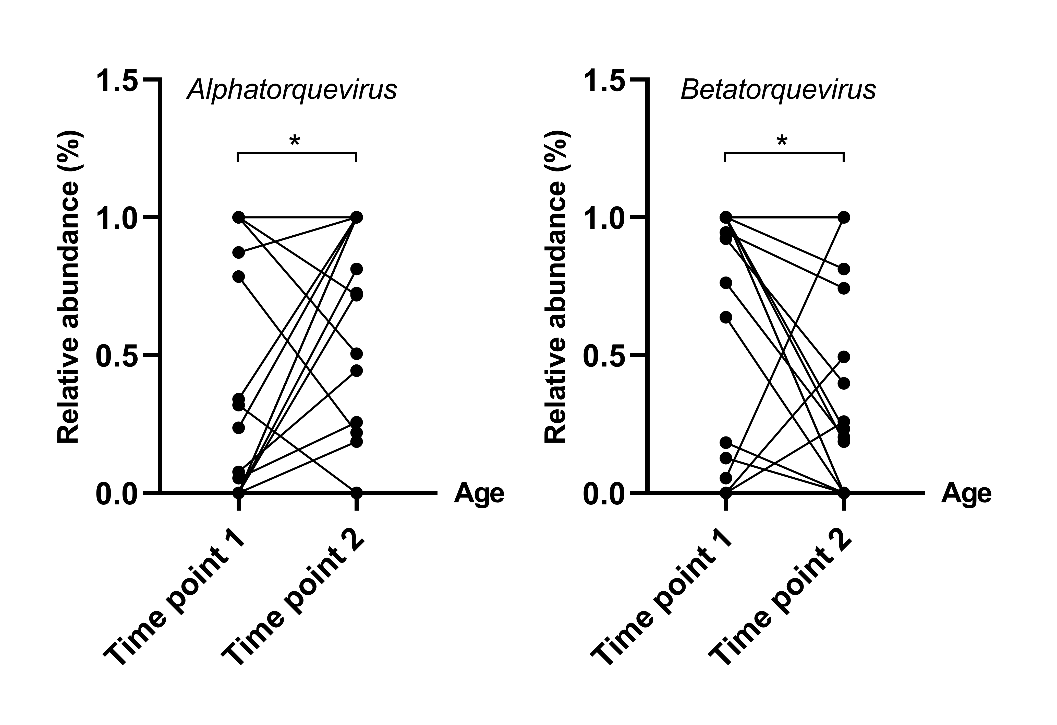


**Figure S2.** The change of the relative abundance of Alphatorquevirus and Betatorquevirus with the age. Only samples that were collected more than 3 months apart were used for the comparison. Wilcoxon matched-pairs rank test was used, * p<0.05.


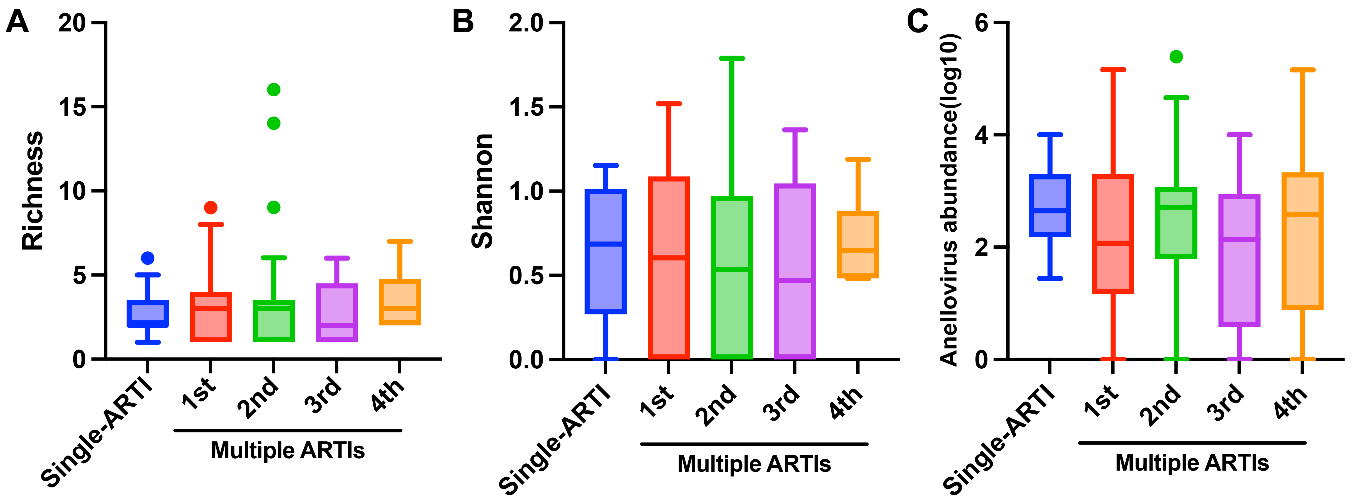
**Figure S3. The anellovirus diversity of different groups.** Comparison of the richness (A), shannon (B), and abundance (C) of anellovirus between single and multiple ARTI groups. Alpha diversity and abundance was calculated based on the results from NCBI annotation using anellovirus contigs. The comparisons were performed using the Kruskal-Wallis test with dunn’s corrections.


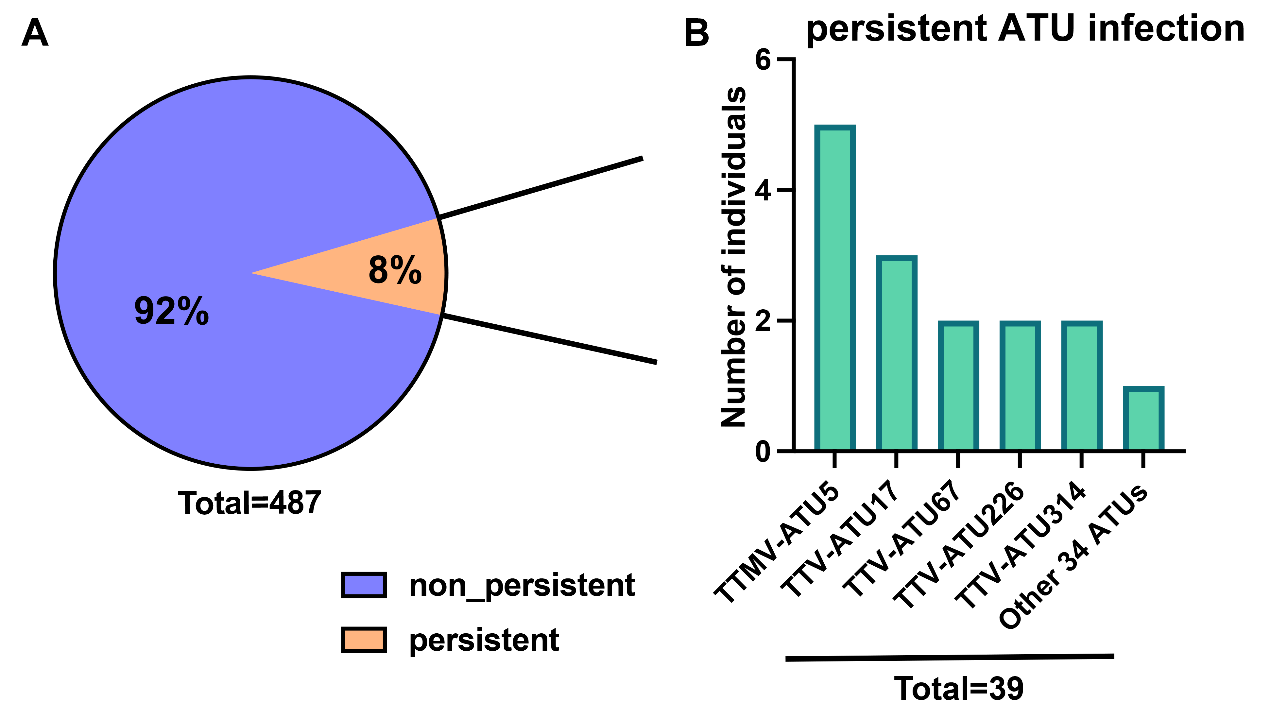


**Figure S4. The distribution of persistent ATUs.** (A) The pie chart shows the percentage of persistent and non-persistent ATUs. (B) Prevalence of the most commonly detected persistent ATUs.
